# Alginate oligosaccharide-induced intestinal morphology, barrier function and epithelium apoptosis modifications have beneficial effects on the growth performance of weaned pigs

**DOI:** 10.1186/s40104-018-0273-x

**Published:** 2018-08-16

**Authors:** Jin Wan, Jiao Zhang, Daiwen Chen, Bing Yu, Xiangbing Mao, Ping Zheng, Jie Yu, Junqiu Luo, Jun He

**Affiliations:** Institute of Animal Nutrition, Sichuan Agricultural University, Chengdu, 611130 Sichuan People’s Republic of China

**Keywords:** Alginate oligosaccharide, Barrier function, Cell apoptosis, Intestinal morphology, Weaned pigs

## Abstract

**Background:**

Alginate oligosaccharide (AOS), produced from alginate by alginate lyase-mediated depolymerisation, is a potential substitute for antibiotics and possesses growth-enhancing effects. Nevertheless, the mechanisms by which AOS regulates porcine growth remain to be elucidated. Therefore, we investigated the AOS-mediated changes in the growth performance of weaned pigs by determining the intestinal morphology, barrier function, as well as epithelium apoptosis.

**Methods:**

Twenty-four weaned pigs were distributed into two groups (*n* = 12) and received either a basal diet (control group) or the same diet supplemented with 100 mg/kg AOS. On d 15, *D*-xylose (0.1 g/kg body weight) was orally administrated to eight randomly selected pigs per treatment, and their serum and intestinal mucosa samples were collected 1 h later.

**Results:**

Our results showed that inclusion of AOS in the diet for 2 wk increased (*P* < 0.05) the average daily body weight gain in weaned pigs. Notably, AOS supplementation ameliorated the intestinal morphology and barrier function, as suggested by the enhanced (*P* < 0.05) intestinal villus height, secretory immunoglobulin A content and goblet cell counts. Compared to the control group, AOS ingestion both decreased (*P* < 0.05) the total apoptotic percentage and increased (*P* < 0.05) the proportion of S phase in the intestinal epithelial cells. Furthermore, AOS not only up-regulated (*P* < 0.05) the B-cell lymphoma-2 (*BCL2*) transcriptional level but also down-regulated (*P* < 0.05) the B-cell lymphoma-2-associated X protein (*BAX*), cysteinyl aspartate-specific proteinase-3 (*caspase-3*) and *caspase-9* transcriptional levels in the small intestine.

**Conclusions:**

In summary, this study provides evidence that supplemental AOS beneficially affects the growth performance of weaned pigs, which may result from the improved intestinal morphology and barrier function, as well as the inhibited enterocyte death, through reducing apoptosis via mitochondria-dependent apoptosis.

## Background

Weaning is one of the most significant events in the life of pigs as they are abruptly forced to adapt to nutritional, immunological and psychological disruptions [1]. The weaning transition of piglets is commonly accompanied by growth retardation and impaired intestinal barrier [2–4]. Studies have also indicated that weaning can disrupt the physiological oxidant and antioxidant equilibrium and lead to oxidative stress [5, 6], eventually inducing epithelium apoptosis and cell cycle arrest in the small intestine of post-weaning piglets [7]. Over the past decades, antibiotic medication has proven an effective preventative and treatment method, used worldwide to treat these issues. However, the widespread use of antibiotics has led, at least in part, to bacterial resistance, resulting in the delayed administration of effective therapy, as well as morbidity and mortality in both humans and animals [8–10]. Hence, numerous antibiotic alternatives have been investigated, among which oligosaccharides have attracted considerable research interest, due to their health benefits in weaned pigs [11, 12].

Alginate, a naturally occurring anionic polysaccharide that is extracted from marine brown algae, is composed of two types of uronic acid monomers, distributed as blocks of 1,4-linked β-*D*-mannuronic acid (M) or α-*L*-guluronic acid (G), as well as heteropolymeric mixed sequences (M–G, usually alternating) [13, 14]. Alginate oligosaccharide (AOS), prepared by depolymerising alginate, is a non-immunogenic, non-toxic, biodegradable polymer with reported multifarious biological properties [15], including anti-oxidation [16], anti-apoptotic [17], anti-inflammatory [18] and anti-tumour effects [19]. These beneficial properties of AOS suggest it may be an effective dietary ingredient, yet the use of AOS as a food supplement for humans or animals is contemporarily still in its infancy. Although emerging evidence identified that AOS supplements favourably enhanced the growth performance in piglets after weaning [20], the AOS mechanisms responsible for this benefit are poorly understood. As such, further elucidation is meaningful and essential.

Accordingly, the present study was performed to explore the effects of AOS supplementation on the intestinal architecture, barrier function and epithelium apoptosis in weaned pigs, aiming to provide partial theoretical evidence for the mechanisms by which AOS enhances growth performance of weaned pigs. It is anticipated that our findings will pave the way for developing AOS as a functional food for both humans and animals in the near future.

## Methods

### Animal care and experimental design

Initially, 24 pigs (Duroc × Landrace × Yorkshire), weaned at 21 d and with an average body weight (BW) of (6.21 ± 0.0 9) kg, were assigned to two treatments with 12 replicates per treatment. The treatment groups including a control group (CON), in which pigs were fed a basal diet, and an AOS group, in which pigs were fed a basal diet supplemented with 100 mg/kg AOS (provided by the Dalian Institute of Chemical Physics, Chinese Academy of Sciences, Dalian, China). The basal diet was formulated to meet or exceed the nutrient requirements recommended by the National Research Council (Table 1) [21]. During the 14-day experimental period, all pigs were individually housed in metabolism cages (0.7 m × 1.5 m) in a temperature- (24−26 °C), humidity- (65% ± 5%) and light-controlled room and were given ad libitum access to feed and water.

**Table 1:** Ingredients and nutrient composition of the basal diet

Ingredient	Content, %	Nutrient composition^c^	Content, %
Corn (7.8% crude protein)	28.80	Digestible energy, MJ/kg	14.85
Extruded corn (7.8% crude protein)	26.00	Crude protein	19.35
Soybean meal (44.2% crude protein)	11.00	Calcium	0.83
Extruded soybean	10.00	Total phosphorus	0.60
Whey powder (low protein)	7.00	Available phosphorus	0.43
Soybean protein concentrate	5.00	Lysine	1.37
Fish meal (62.5% crude protein)	4.00	Methionine	0.49
Sucrose	4.00	Methionine + Cysteine	0.76
Soybean oil	1.50	Threonine	0.81
Limestone	0.75	Tryptophan	0.22
Dicalcium phosphate	0.60		
*L*-Lysine-HCl (78%)	0.40		
NaCl	0.30		
*DL*-Methionine	0.18		
*L*-Threonine (98.5%)	0.10		
Chloride choline	0.10		
Tryptophan (98%)	0.02		
Vitamin premix^a^	0.05		
Mineral premix^b^	0.20		
Total	100		

^a^The vitamin premix provided the following per kg of diets: 6,000 IU vitamin (V) A, 3,000 IU VD_3_, 24 mg VE, 3 mg VK_3_, 1.5 mg VB_1_, 6 mg VB_2_, 3 mg VB_6_, 0.02 mg VB_12_, 14 mg niacin, 15 mg pantothenic acid, 1.2 mg folic acid and 0.15 mg biotin

^b^The mineral premix provided the following per kg of diets: 100 mg Fe, 6 mg Cu, 100 mg Zn, 4 mg Mn, 0.30 mg I and 0.35 mg Se

^c^Values are calculated composition

### Growth performance assessment

At the start and end of the experiment, the pigs were individually weighed before feeding, and daily feed consumption per pig was measured throughout the study. Growth performance indices, including average daily body weight gain (ADG), average daily feed intake (ADFI) and the gain-to-feed ratio (G:F), were subsequently determined for each group from the data obtained.

### Sample collection

On the morning of d 15, after overnight starvation, eight pigs from each treatment were randomly selected and orally infused with *D*-xylose at the dose of 0.1 g/kg BW [22, 23]. After infusion of *D*-xylose (1 h), blood samples were collected by jugular vein puncture and placed in 10-mL vacuum tubes (non-anticoagulant). The samples were centrifuged at 3,500×*g*, 4 °C for 15 min, to acquire serum, and stored at −20 °C, until measurement of *D*-xylose concentration.

After blood sampling, the same pigs were anaesthetised with an intravenous injection of sodium pentobarbital (200 mg/kg BW), and the tissues of the duodenum, jejunum and ileum were immediately isolated [24]. Approximately 5-cm duodenal, jejunal and ileal middle segments were gently flushed with ice-cold phosphate buffered saline (PBS), followed by fixation in PBS for flow cytometry or in 4% paraformaldehyde solution for morphological and immunohistochemical analyses. Finally, the residual duodenal, jejunal and ileal segments were scraped with a scalpel blade, and the collected mucosa stored at −80 °C for quantitative real-time polymerase chain reaction (qPCR) analysis.

### Serum *D*-xylose determination

Serum *D*-xylose was quantitated using a *D*-xylose assay kit (Nanjing Jiancheng Bioengineering Institute, Nanjing, China), by following the manufacturer’s protocols. The absorbance of the reaction mixture was acquired spectrophotometrically at 554 nm, using a multi-mode microplate reader (SpectraMax M2, Molecular Devices, Sunnyvale, CA, USA). *D*-Xylose concentration was presented as milligrams per litre of serum (mg/L).

### Histomorphological analysis and cell counting

One-cm long duodenal, jejunal and ileal samples were dehydrated through a graded series of ethanol and embedded in paraffin. Cross-sections of each sample were prepared, stained with haematoxylin and eosin (H&E), and then sealed with neutral resin. Ultrathin sections of the duodenal, jejunal and ileal samples were examined for villus height, villus width and crypt depth, using an image processing and analysis system (Image-Pro Plus 6.0, Media Cybernetics, Inc., Bethesda, MD, USA). Afterwards, the goblet cell and columnar cell counts per villus were also assessed. Villus height was recorded as the distance from the tip of the villi to the villus-crypt junction, and width was measured at half of the villus height [25]. Crypt depth was expressed as the invaginated depth between adjacent villi. A total of 10 intact, well-oriented, crypt-villus units were analysed in triplicate per intestinal segment. The values obtained from 10 villi, in triplicate by each intestinal segment, were averaged. The villus height-to-crypt depth ratio was computed from the measurements obtained above, and the villus surface area (mm^2^) was calculated by multiplying 2π(villus width/2) by the villus height [26].

### Immunohistochemistry

For immunohistochemistry, the paraformaldehyde-fixed duodenal, jejunal and ileal samples were embedded in paraffin and sectioned into 2 μm thickness, then collected on glass slides. After deparaffinisation and hydration, the sections were pre-treated with 3% H_2_O_2_ in methanol at room temperature for 10 min, to quench endogenous peroxidase activity and, then, heated in 10 mmol/L citrate buffer (pH 6.0) to retrieve the antigen. After several rinses in PBS, the sections were blocked with 10% goat serum at room temperature for 20 min, to eliminate non-specific antibody binding and then incubated overnight at 4 °C with 1:200 dilution of rabbit anti-secretory immunoglobulin A (sIgA) antibody (Beijing Biosynthesis Biotechnology Co., Ltd., Beijing, China). After rinsing with PBS several times, the sections were incubated with biotinylated goat anti-rabbit IgG secondary antibody (Beijing Zhongshan Golden Bridge Biotechnology Co., Ltd., Beijing, China) at 37 °C for 30 min. After rinsing several times in PBS, immunodetection was conducted, using 3,3′-diaminobenzidine (DAB) as the chromogen. The sections were counterstained with haematoxylin and mounted in neutral resin. For each section in the Motic BA210 digital microscope (Motic China Group Co., Ltd., Xiamen, China), five fields of vision were randomly selected, with a fixed window area. The integrated optical density of sIgA in the duodenal, jejunal and ileal mucosa was detected by using Image-Pro Plus 6.0 image analysis system (Media Cybernetics, Inc), and the sIgA protein expression was reflected by the mean value of the integrated optical density.

### Enterocyte apoptosis detection

Duodenal, jejunal and ileal epithelial cells were isolated, to measure the proportion of apoptotic cells by flow cytometry with a PE Annexin V Apoptosis Detection Kit I (Becton, Dickinson and Company, BD Biosciences, San Jose, CA, USA) [27]. Briefly, the excised mucosal layer of the duodenum, jejunum and ileum were isolated, and then, ground and filtered to form a cell suspension. The cells were carefully washed twice with ice-cold PBS and suspended in the PBS at 1 × 10^6^ cells/mL. After adding 5 μL of PE Annexin V and 5 μL of 7-aminoactinomycin D (7-AAD) to a 100-μL aliquot of the cell suspension, the mixture was incubated at room temperature for 15 min in a dark room. Afterwards, 400 μL of Annexin V Binding Buffer (1×) was added, and the apoptotic cells were examined by flow cytometry (CytoFlex, Beckman Coulter, Inc., Brea, CA, USA) within 1 h.

### Enterocyte cell cycle analysis

For enterocyte cell cycle analysis, duodenal, jejunal and ileal epithelial cell suspensions were prepared, as described above for apoptosis detection. A total 1 mL of cell suspension was transferred to a 5-mL culture tube. After adding 1 mL of 0.25% Triton X-100, the mixture was incubated at 4 °C for 10 min, and the cells washed with PBS. Next, 5 μL of 7-AAD was added to 100 μL of cell suspension and incubated at 4 °C for 30 min in the dark. Finally, 400 μL of PBS was added. The cell cycle distribution was assayed using a CytoFlex flow cytometer (Beckman Coulter, Inc) within 45 min and analysed by ModFit LT 5.0 (Verity Software House, Topsham, ME, USA) [28]. The proliferating index (%) was calculated by the formula $\frac{\mathrm{S}+\left({\mathrm{G}}_2+\mathrm{M}\right)}{\left({\mathrm{G}}_0/{\mathrm{G}}_1\right)+\mathrm{S}+\left({\mathrm{G}}_2+\mathrm{M}\right)}$× 100.

### Total RNA isolation and reverse transcription

Frozen duodenal, jejunal and ileal samples (about 0.1 g), respectively, were pulverised in liquid nitrogen and subsequently homogenised in 1 mL of RNAiso Plus (Takara Biotechnology Co., Ltd., Dalian, China) to extract total RNA, according to the manufacturer’s instructions. The concentration and quality of total RNA were assessed using a spectrophotometer (NanoDrop 2000, Thermo Fisher Scientific, Inc., Waltham, MA, USA), considering the high-quality absorbance ratio (260/280 nm) being within 1.8 and 2.0, and the integrity of total RNA was checked by electrophoresis on a 1% agarose gel. Next, a volume equivalent to 1 μg total RNA of each duodenal, jejunal and ileal sample, respectively, was used to synthesise cDNA, based on the protocol of PrimeScript™ RT reagent kit with gDNA Eraser (Takara Biotechnology Co., Ltd). The synthesis was achieved in two steps: 37 °C for 15 min, followed by 85 °C for 5 s.

### qPCR

Mucin 1 (*MUC1*), *MUC2*, *MUC4*, B-cell lymphoma-2-associated X protein (*BAX*), B-cell lymphoma-2 (*BCL2*), *FAS*, cysteinyl aspartate-specific proteinase-3 (*caspase-3*), *caspase-8* and *caspase-9* mRNA levels in intestinal mucosa were quantified using qPCR, as described by Wan et al. [29]. In brief, the specific primers were designed using Primer Express 3.0 software (Applied Biosystems, Foster City, CA, USA) and purchased from Sangon Biotech Co., Ltd. (Shanghai, China), as depicted in Table 2. All qPCR reactions were performed in triplicate on a QuanStudio™ 6 Flex Real-Time PCR System (Applied Biosystems), using SYBR® Premix Ex Taq™ II (Tli RNaseH Plus) (Takara Biotechnology Co., Ltd). Amplification was performed in a final volume of 10 μL, which consisted of 5 μL of SYBR Premix Ex Taq II (Tli RNaseH Plus, 2×), 0.2 μL ROX Reference Dye II (50×), 0.4 μL forward primer (10 μmol/L), 0.4 μL reverse primer (10 μmol/L), 1 μL cDNA and 3 μL diethylpyrocarbonate-treated water, under the following cycling conditions: 95 °C for 30 s, followed by 40 cycles: at 95 °C for 5 s and 60 °C for 34 s. After the amplification phase, a melt curve analysis was performed at 95 °C for 15 s, 60 °C for 1 min and 95 °C for 15 s, to confirm the specificity of the amplification reaction. Porcine glyceraldehyde-3-phosphate dehydrogenase (*GAPDH*) gene was chosen as the housekeeping gene, to normalise the expression levels of the target genes. Amplification efficiencies were calculated from the specific gene standard curves that were generated from 10-fold serial dilutions, quantifying six concentrations. After verification that the primers amplified with an efficiency of approximately 100%, the relative gene expressions between the two groups were calculated, based on the 2^-ΔΔCt^ method [30].

**Table 2:** Primer sequences for quantitative real-time polymerase chain reaction

Gene^a^	Primer sequence (5′→3′)	Size, bp	Accession No.
*MUC1*	Forward: GTGCCGCTGCCCACAACCTG Reverse: AGCCGGGTACCCCAGACCCA	141	XM_001926883.4
*MUC2*	Forward: GGTCATGCTGGAGCTGGACAGT Reverse: TGCCTCCTCGGGGTCGTCAC	181	XM_013989745.1
*MUC4*	Forward: GATGCCCTGGCCACAGAA Reverse: TGATTCAAGGTAGCATTCATTTGC	89	XM_001926442.1
*BAX*	Forward: CTGACGGCAACTTCAACTGG Reverse: CGTCCCAAAGTAGGAGAGGA	200	XM_003127290.4
*BCL2*	Forward: AGCATGCGGCCTCTATTTGA Reverse: GGCCCGTGGACTTCACTTAT	120	XM_003121700.2
*FAS*	Forward: TGATGCCCAAGTGACTGACC Reverse: GCAGAATTGACCCTCACGAT	103	NM_213839.1
*caspase-3*	Forward: GTGGGACTGAAGATGACA Reverse: ACCCGAGTAAGAATGTG	190	NM_214131.1
*caspase-8*	Forward: AGACAAGGGCATCATCATCGG Reverse: GGTTTACCAAGAAGGGAACGG	102	NM_001031779.2
*caspase-9*	Forward: AATGCCGATTTGGCTTACGT Reverse: CATTTGCTTGGCAGTCAGGTT	195	XM_003127618.4
*GAPDH*	Forward: ATGGTGAAGGTCGGAGTGAAC Reverse: CTCGCTCCTGGAAGATGGT	235	NM_001206359.1

^a^*MUC1*, mucin 1; *MUC2*, mucin 2; *MUC4*, mucin 4; *BAX*, B-cell lymphoma-2-associated X protein; *BCL2*, B-cell lymphoma-2; *caspase-3*, cysteinyl aspartate-specific proteinase-3; *caspase-8*, cysteinyl aspartate-specific proteinase-8; *caspase-9*, cysteinyl aspartate-specific proteinase-9; *GAPDH*, glyceraldehyde-3-phosphate dehydrogenase

### Statistical analysis

All data were analysed by a Student’s *t*-test using SAS 9.0 (SAS Inst., Inc., Cary, NC, USA). Each pig served as a statistical unit. Data are shown as the mean ± standard error. *P* < 0.05 was considered significant when used to compare the differences between the CON group and the AOS group.

## Results

### Growth performance

Although AOS addition did not have a significant effect (*P* > 0.05) on G:F, the ADG and ADFI were elevated (*P* < 0.05) by supplemental AOS throughout the entire experimental period (Table 3).

**Table 3:** Effects of alginate oligosaccharide on the growth performance of weaned pigs throughout the entire experimental period^a^

Item^c^	Treatment^b^		*P*-value
	CON	AOS	
Initial BW, kg	6.20 ± 0.09	6.21 ± 0.08	0.973
Final BW, kg	8.73 ± 0.16	9.46 ± 0.20^**^	0.009
D 1−14			
ADG, g/d	180.36 ± 9.70	232.44 ± 13.51^**^	0.005
ADFI, g/d	253.12 ± 11.75	311.61 ± 19.64^*^	0.018
G:F	0.72 ± 0.02	0.75 ± 0.02	0.213

^*^P < 0.05 versus the CON group. ^**^ *P* < 0.01 versus the CON group

^a^Values are the means of 12 replicates per treatment

^b^CON, control (a corn-soybean basal diet); AOS, alginate oligosaccharide (the basal diet supplemented with 100 mg/kg alginate oligosaccharide)

^c^BW, body weight; ADG, average daily body weight gain; ADFI, average daily feed intake; G:F, the gain-to-feed ratio

### Serum *D*-xylose concentration

**Fig. 1:**
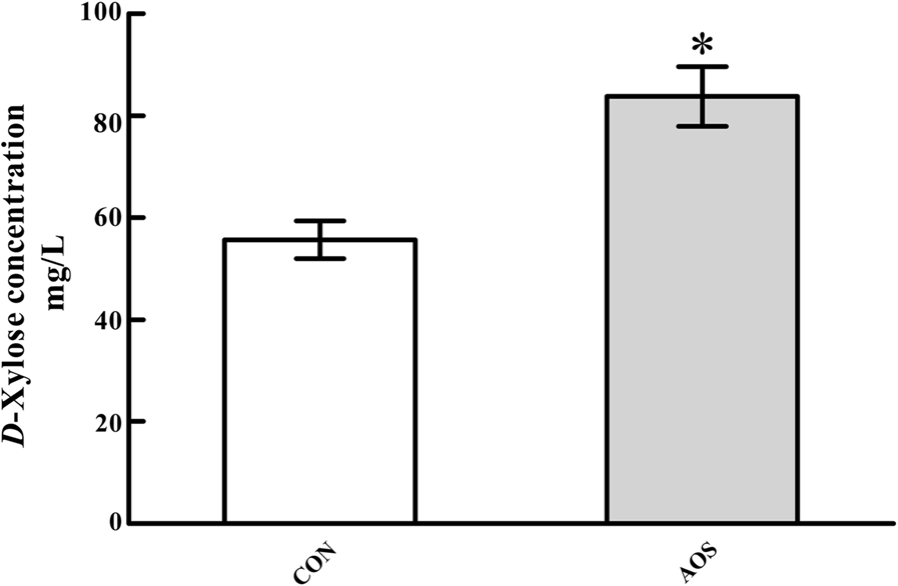
Effects of alginate oligosaccharide on the serum *D*-xylose concentration of weaned pigs. Values are means (8 pigs/treatment), with standard errors represented by vertical bars. ^*^P < 0.05 (indicates that the serum *D*-xylose concentration is significantly higher in the AOS group than CON group). CON, control (a corn-soybean basal diet); AOS, alginate oligosaccharide (the basal diet supplemented with 100 mg/kg alginate oligosaccharide)

Fig. 1 reveals the effects of AOS supplementation on the serum *D*-xylose level in weaned pigs. The data showed that the pigs in the AOS group had a higher (*P* < 0.05) serum *D*-xylose concentration compared to the CON group.

### Intestinal architecture

**Fig. 2:**
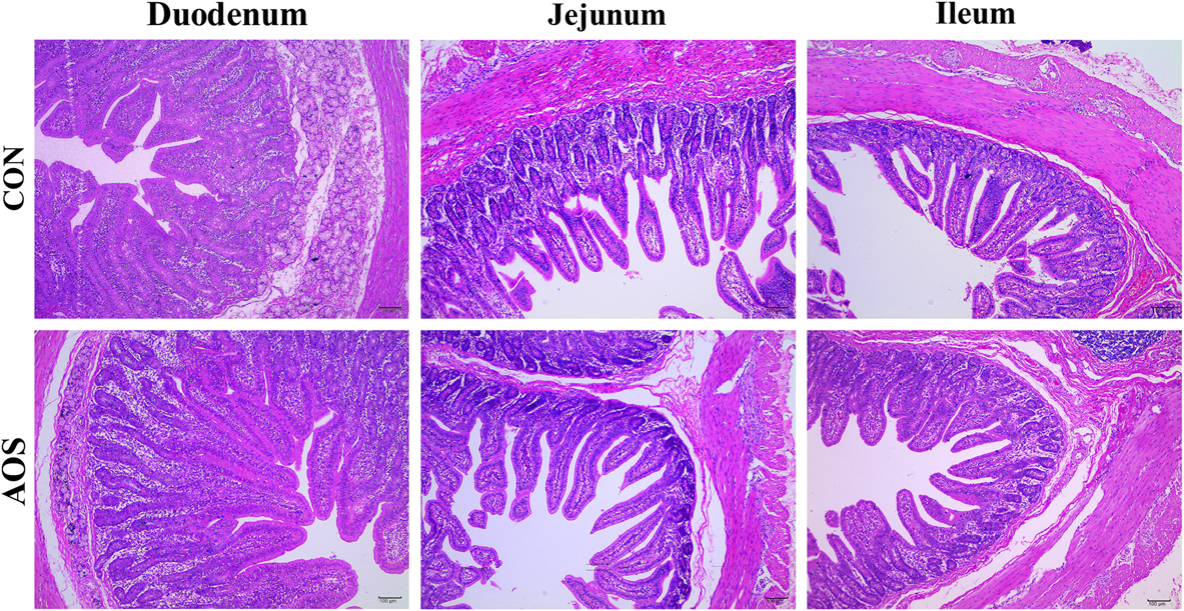
Histological evaluation of the small intestinal tissues after exposure to alginate oligosaccharide (H&E; × 100). CON, control (a corn-soybean basal diet); AOS, alginate oligosaccharide (the basal diet supplemented with 100 mg/kg alginate oligosaccharide). Scale bar is 100 μm

H&E staining of the small intestine tissues after exposure to AOS indicated that AOS supplementation caused duodenal and jejunal architecture alternations but failed to change the ileal structure (Fig. 2).

Next, the specific duodenal, jejunal and ileal morphological parameters for the two groups were calculated (Table 4). Dietary AOS inclusion resulted in a significant increase (*P* < 0.05) in the villus height and the villus height-to-crypt depth ratio in both, the duodenum and jejunum, as well as the jejunal villus surface area. Moreover, there were no significant differences (*P* > 0.05) in the ileal morphological parameters between the two treatments.

**Table 4:** Effects of alginate oligosaccharide on the intestinal mucosal morphology of weaned pigs^a^

Item	Treatment^b^		*P*-value
	CON	AOS	
Duodenum			
Villus height, μm	407.63 ± 11.36	457.88 ± 17.07^*^	0.028
Villus width, μm	132.26 ± 5.62	136.98 ± 7.61	0.626
Crypt depth, μm	224.74 ± 4.17	226.91 ± 8.58	0.823
Villus surface area, mm^2^	0.17 ± 0.01	0.20 ± 0.01	0.054
Villus height:Crypt depth	1.81 ± 0.03	2.03 ± 0.08^*^	0.030
Jejunum			
Villus height, μm	408.75 ± 10.49	456.94 ± 12.07^**^	0.009
Villus width, μm	109.22 ± 3.61	114.36 ± 5.18	0.429
Crypt depth, μm	194.80 ± 1.95	189.25 ± 2.43	0.096
Villus surface area, mm^2^	0.14 ± 0.01	0.16 ± 0.01^*^	0.031
Villus height:Crypt depth	2.10 ± 0.06	2.41 ± 0.04^**^	< 0.001
Ileum			
Villus height, μm	334.83 ± 2.86	351.34 ± 7.73	0.077
Villus width, μm	113.17 ± 5.19	123.39 ± 5.07	0.180
Crypt depth, μm	169.61 ± 5.19	174.41 ± 6.70	0.580
Villus surface area, mm^2^	0.12 ± 0.01	0.14 ± 0.01	0.120
Villus height:Crypt depth	2.01 ± 0.05	2.04 ± 0.09	0.793

^*^P < 0.05 versus the CON group. ^**^P < 0.01 versus the CON group

^a^Values are the means of 8 replicates per treatment

^b^CON, control (a corn-soybean basal diet); AOS, alginate oligosaccharide (the basal diet supplemented with 100 mg/kg alginate oligosaccharide)

### Goblet and columnar cell counts

A summary of the goblet and columnar cell counts after AOS supplementation is provided in Table 5. AOS supplementation did not affect (*P* > 0.05) the columnar cell counts but increased (*P* < 0.05) the goblet cell counts in the duodenum and jejunum. There was no impact (*P* > 0.05) on the ileal goblet and columnar cell counts by AOS ingestion.

**Table 5:** Effects of alginate oligosaccharide on the intestinal goblet and columnar cell counts of weaned pigs^a^

Item	Treatment^b^		*P*-value
	CON	AOS	
Duodenum (number/villus)			
Goblet cells	8.69 ± 0.26	10.03 ± 0.43^*^	0.018
Columnar cells	71.94 ± 3.10	75.09 ± 3.53	0.514
Jejunum (number/villus)			
Goblet cells	7.31 ± 0.34	9.79 ± 0.23^**^	< 0.001
Columnar cells	70.25 ± 1.57	73.55 ± 1.51	0.153
Ileum (number/villus)			
Goblet cells	11.33 ± 0.79	11.60 ± 0.37	0.758
Columnar cells	78.20 ± 1.77	81.91 ± 2.40	0.233

^*^P < 0.05 versus the CON group. ^**^P < 0.01 versus the CON group

^a^Values are the means of 8 replicates per treatment

^b^CON, control (a corn-soybean basal diet); AOS, alginate oligosaccharide (the basal diet supplemented with 100 mg/kg alginate oligosaccharide)

### sIgA content

**Fig. 3:**
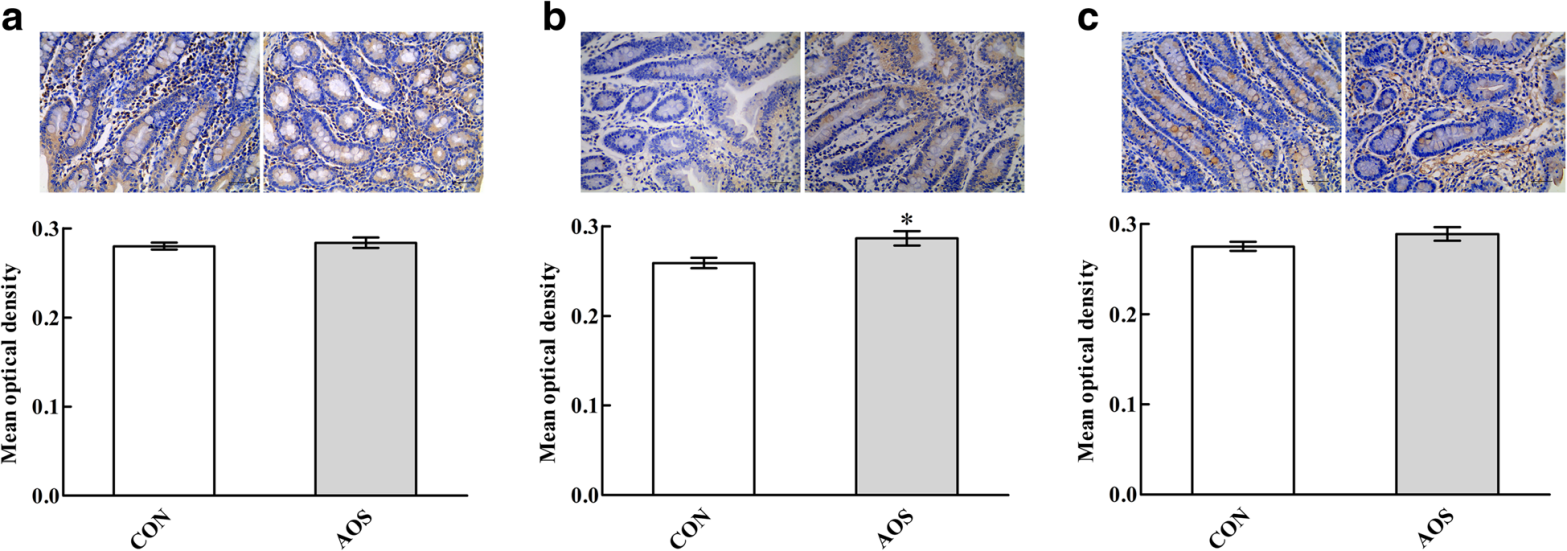
Effects of alginate oligosaccharide on the sIgA content in the duodenum (**a**), jejunum (**b**) and ileum (**c**) of weaned pigs (immunohistochemistry; × 400). Values are means (8 pigs/treatment), with standard errors represented by vertical bars. ^*^P < 0.05 (indicates that the sIgA content is significantly higher in the AOS group than CON group). CON, control (a corn-soybean basal diet); AOS, alginate oligosaccharide (the basal diet supplemented with 100 mg/kg alginate oligosaccharide). sIgA, secretory immunoglobulin A. Scale bar is 40 μm

Fig. 3 presents the mean optical density of intestinal sIgA in the CON and AOS groups. Interestingly, the jejunal mean optical density of sIgA was higher (*P* < 0.05) in the AOS group than CON group, whereas the duodenal and ileal mean optical densities of sIgA were not affected (*P* > 0.05) by AOS supplementation.

### Apoptotic percentage

**Fig. 4:**
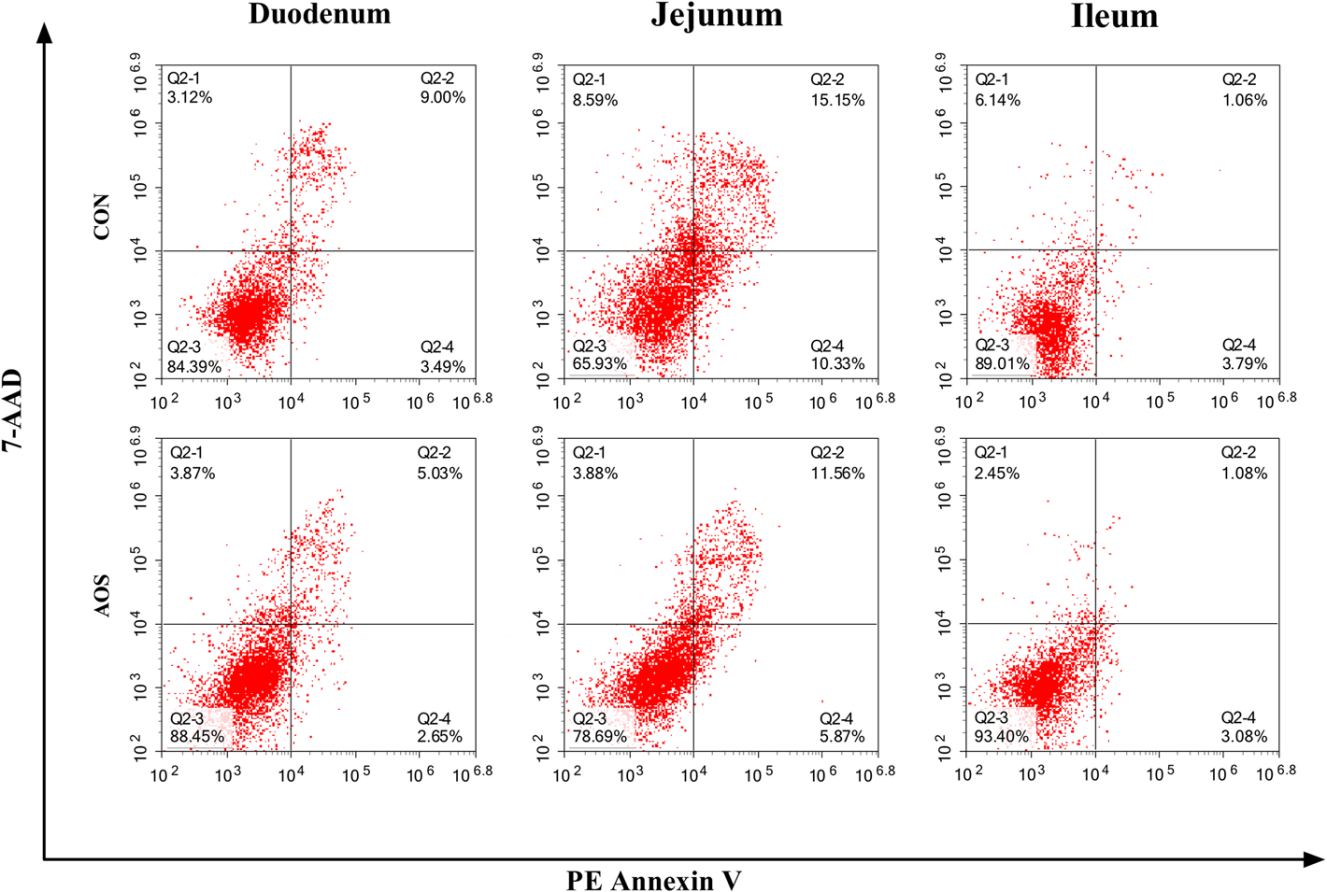
Percentage of apoptotic cells in the small intestine of weaned pigs fed diets containing or lacking alginate oligosaccharide. Frames were divided into four quadrants: Q2–1 represents necrotic cells; Q2–2 represents late-stage apoptotic cells; Q2–3 represents normal cells; Q2–4 represents early-stage apoptotic cells. CON, control (a corn-soybean basal diet); AOS, alginate oligosaccharide (the basal diet supplemented with 100 mg/kg alginate oligosaccharide). 7-AAD, 7-aminoactinomycin D

The impacts of AOS on the intestinal epithelial cell apoptosis are demonstrated in Fig. 4 and Table 6. Compared to the control group, AOS supplementation decreased (*P* < 0.05) the early- and late-stage apoptotic cell percentages, as well as the total apoptotic cells percentage, in the jejunal epithelium. Furthermore, there were no marked differences (*P >* 0.05) in the duodenal and ileal epithelial cell apoptotic percentages between the AOS and CON groups.

**Table 6:** Effects of alginate oligosaccharide on the enterocyte apoptosis of weaned pigs^a^

Item	Treatment^b^		*P*-value
	CON	AOS	
Duodenum, %			
Early-stage apoptotic cells	2.94 ± 0.31	2.98 ± 0.22	0.909
Late-stage apoptotic cells	9.31 ± 1.18	5.92 ± 0.46	0.055
Total apoptotic cells	12.25 ± 1.32	8.90 ± 0.61	0.083
Jejunum, %			
Early-stage apoptotic cells	10.98 ± 0.99	6.31 ± 0.68^*^	0.018
Late-stage apoptotic cells	15.70 ± 0.85	10.86 ± 1.02^*^	0.022
Total apoptotic cells	26.68 ± 0.61	17.17 ± 0.35^**^	< 0.001
Ileum, %			
Early-stage apoptotic cells	3.83 ± 0.46	2.93 ± 0.34	0.149
Late-stage apoptotic cells	1.14 ± 0.18	1.03 ± 0.11	0.618
Total apoptotic cells	4.97 ± 0.42	3.96 ± 0.32	0.093

^*^P < 0.05 versus the CON group. ^**^P < 0.01 versus the CON group

^a^Values are the means of 8 replicates per treatment

^b^CON, control (a corn-soybean basal diet); AOS, alginate oligosaccharide (the basal diet supplemented with 100 mg/kg alginate oligosaccharide)

### Cell cycle distribution

**Fig. 5:**
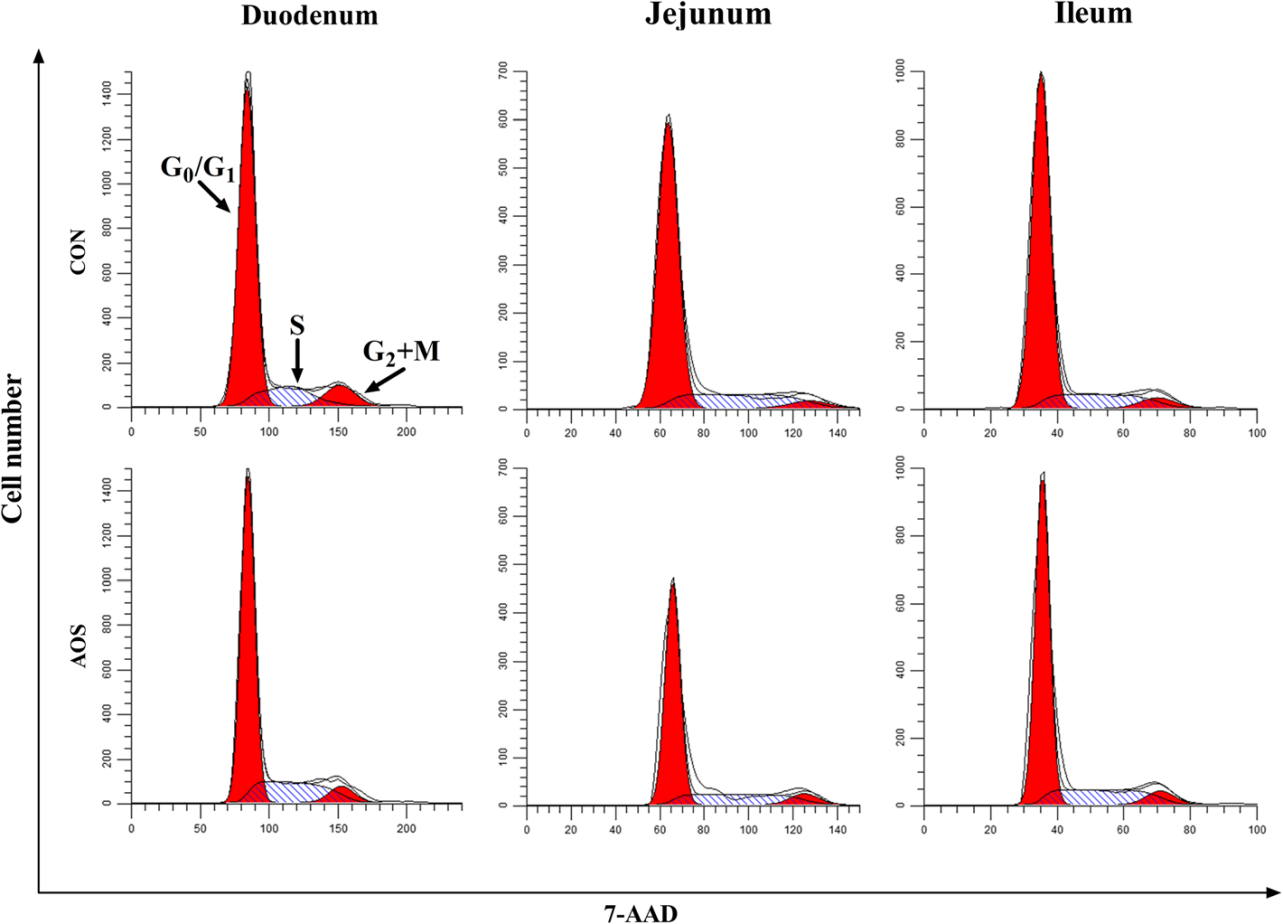
DNA histogram of the cell cycle in the small intestinal epithelium of weaned pigs fed diets containing or lacking alginate oligosaccharide. The first peak in the DNA histogram of the small intestinal epithelium cell cycle is in G_0_/G_1_ phase, the second peak is in G_2_ + M phase, and S phase lies between these two peaks. CON, control (a corn-soybean basal diet); AOS, alginate oligosaccharide (the basal diet supplemented with 100 mg/kg alginate oligosaccharide). 7-AAD, 7-aminoactinomycin D

Fig. 5 and Table 7 demonstrate that AOS supplementation decreased (*P* < 0.05) the proportion of G_0_/G_1_ phase cells but increased (*P* < 0.05) the ratio of S phase cells, as well as the proliferating index, in the jejunal epithelium. Furthermore, the duodenal and ileal cell cycle distributions did not markedly change (*P >* 0.05) after AOS supplementation.

**Table 7:** Effects of alginate oligosaccharide on the enterocyte proliferation of weaned pigs^a^

Item	Treatment^b^		*P*-value
	CON	AOS	
Duodenum, %			
G_0_/G_1_ phase cells	75.15 ± 1.41	70.65 ± 1.37	0.051
S phase cells	18.71 ± 1.25	22.05 ± 0.78	0.053
G_2_ + M phase cells	6.02 ± 1.05	6.54 ± 0.72	0.698
Proliferating index	24.77 ± 1.39	28.81 ± 1.28	0.064
Jejunum, %			
G_0_/G_1_ phase cells	75.93 ± 1.81	70.42 ± 0.78^*^	0.023
S phase cells	16.66 ± 1.37	21.88 ± 0.87^*^	0.012
G_2_ + M phase cells	5.98 ± 0.74	7.62 ± 0.55	0.112
Proliferating index	22.99 ± 1.51	29.53 ± 0.79^**^	0.005
Ileum, %			
G_0_/G_1_ phase cells	73.57 ± 2.04	68.74 ± 1.98	0.128
S phase cells	17.78 ± 0.90	21.64 ± 1.43	0.052
G_2_ + M phase cells	8.10 ± 1.05	9.13 ± 1.11	0.521
Proliferating index	26.04 ± 1.90	30.89 ± 2.27	0.140

^*^P < 0.05 versus the CON group. ^**^P < 0.01 versus the CON group

^a^Values are the means of 8 replicates per treatment

^b^CON, control (a corn-soybean basal diet); AOS, alginate oligosaccharide (the basal diet supplemented with 100 mg/kg alginate oligosaccharide)

### Mucins gene expressions

**Fig. 6:**
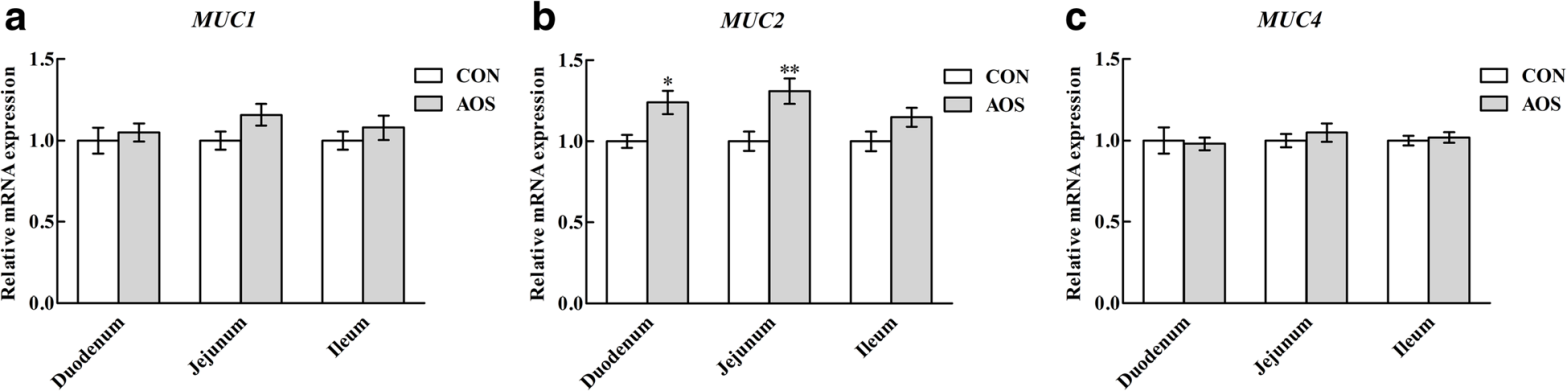
Relative mRNA abundances of *MUC1* (**a**), *MUC2* (**b**) and *MUC4* (**c**) in the small intestine of weaned pigs fed diets containing or lacking alginate oligosaccharide. Values are means (8 pigs/treatment), with standard errors represented by vertical bars. ^*^P < 0.05 or ^**^P < 0.01 (indicates that the gene mRNA levels between the AOS and CON groups differ significantly). CON, control (a corn-soybean basal diet); AOS, alginate oligosaccharide (the basal diet supplemented with 100 mg/kg alginate oligosaccharide). *MUC1*, mucin 1; *MUC2*, mucin 2; *MUC4*, mucin 4

According to Fig. 6, pigs supplemented with AOS had an increase (*P* < 0.05) in mucin 2 (*MUC2*) transcription in the duodenal and ileal mucosae, but not (*P >* 0.05) in the ileal mucosa. Besides, no effects (*P >* 0.05) were detected on the *MUC1* and *MUC4* transcriptions in all of the three intestinal mucosae after AOS ingestion.

### Apoptosis-related genes expression

**Fig. 7:**
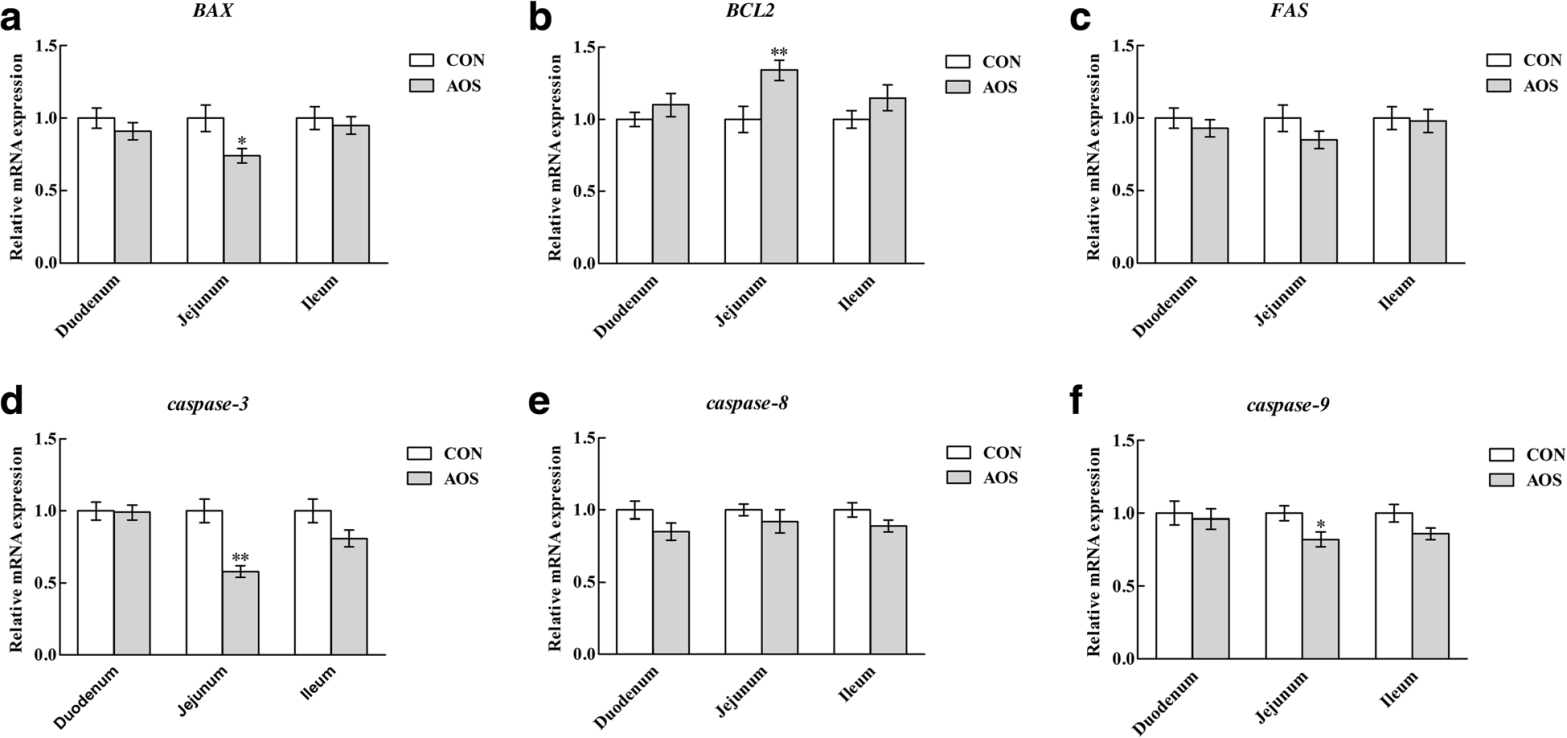
Relative mRNA abundances of *BAX* (**a**), *BCL2* (**b**), *FAS* (**c**), c*aspase-3* (**d**), *caspase-8* (**e**) and *caspase-9* (**f**) in the small intestine of weaned pigs fed diets containing or lacking alginate oligosaccharide. Values are means (8 pigs/treatment), with standard errors represented by vertical bars. ^*^P < 0.05 or ^**^P < 0.01 (indicates that the gene mRNA levels between the AOS and CON groups differ significantly). CON, control (a corn-soybean basal diet); AOS, alginate oligosaccharide (the basal diet supplemented with 100 mg/kg alginate oligosaccharide). *BAX*, B-cell lymphoma-2-associated X protein; *BCL2*, B-cell lymphoma-2; *caspase*-*3*, cysteinyl aspartate-specific proteinase-3; *caspase*-*8*, cysteinyl aspartate-specific proteinase-8; *caspase-9*, cysteinyl aspartate-specific proteinase-9

The transcriptional levels of apoptosis-related genes in the small intestine are illustrated in Fig. 7. Compared to the CON group, AOS ingestion decreased (*P* < 0.05) the pro-apoptotic factor *BAX*, *caspase*-*3* and *caspase*-*9* mRNA abundances and increased (*P* < 0.05) the anti-apoptotic factor *BCL2* mRNA abundance in the jejunal mucosa, but not (*P >* 0.05) in the duodenal and ileal mucosa. However, no difference (*P >* 0.05) was observed in the *FAS* and *caspase*-*8* mRNA abundances among the three intestinal mucosae, after AOS supplementation.

## Discussion

Compromising alterations in intestinal architecture, such as villus atrophy and crypt hyperplasia, are commonly encountered in post-weaning piglets [31, 32]. However, a decrease in the villus height-to-crypt depth ratio or a reduced villus surface area is considered deleterious for digestion and absorption and could lead to retarded growth in post-weaning piglets [33, 34]. Consequently, maintaining the normal intestinal architecture and function is essential for growth and development in piglets after weaning [35]. It is therefore noteworthy that an increased villus height-to-crypt depth ratio in the duodenum and jejunum, as well as an increased villus surface area in the jejunum, was seen in AOS-supplemented pigs. These observations support the notion that AOS inclusion in the diet can change the intestinal morphological structure, and thereby promote the intestinal digestion-absorption function in piglets after weaning [36]. Meanwhile, the increased entry of orally administered *D*-xylose into the blood after AOS ingestion further corroborates the aforementioned view [37]. These findings are sufficient to suggest that the growth-promoting effects of AOS on weaned pigs can be partially attributable to the improved intestinal morphology and function.

There is plentiful of evidence that the early weaning process is correlated with impaired intestinal barrier function in piglets [38, 39]. Interestingly, dietary supplementation with some oligosaccharides provides a promising approach to improve the intestinal barrier function in weaned pigs [40, 41]. Therefore, we expected that AOS would have benefits on intestinal barrier function when administered to weaned pigs. In the present study, AOS supplementation increased the jejunal mucosal sIgA content, suggesting that dietary inclusion of AOS could enhance the intestinal immune barrier function in weaned pigs [42, 43]. Goblet cells are specialised cells found along the crypt–villus axis of the small intestine that biosynthesis, assemble and secrete mucins (including MUC1, MUC2 and MUC4), which contribute to the mucus layer in the intestine, providing an intestinal chemical barrier function [44–46]. In the current study, more goblet cells in the duodenum and jejunum were noticed after AOS addition, accompanied by an up-regulated *MUC2* transcriptional level in the duodenum and jejunum, indicating that AOS supplementation also improved the intestinal chemical barrier function in weaned pigs. Together, these results revealed that AOS is conducive for repairing weaning-associated intestinal barrier dysfunction in piglets and then possibly improved growth performance.

Apoptosis is a form of physiological cell death, important in controlling the epithelial turnover in the intestinal mucosa. However, dysregulated or excessive apoptosis results in severe intestinal pathology [47]. A recent research certified that weaning could increase enterocyte apoptosis in piglets [48]. Here, we noted that apoptosis was less prevalent in the jejunal epithelial cells in the AOS group than control group, suggesting that AOS may have a protective influence against enterocyte apoptosis promoted by weaning of piglets. In addition to inducing enterocyte apoptosis, weaning also inhibits intestinal epithelial cell proliferation in piglets [49]. Here, we identified that AOS supplementation increased jejunal epithelial cell proliferation, through promoting the transition from G_0_/G_1_ to S phase of the cell cycle. As such, it was confirmed that AOS could alleviate the elevated apoptosis and depressed proliferation of intestinal epithelial cells in piglets caused by weaning and consequently mitigate weaning-induced intestinal structural injury. So far, the molecular mechanisms by which AOS inhibits enterocyte apoptosis in weaned pigs remain unclear. Therefore, we studied the effects of AOS addition in the diet, on signalling molecules involved in enterocyte apoptosis in weaned pigs.

It is well-known that the intrinsic (mitochondrial pathway) and extrinsic (cytoplasmic pathway) pathways are two major apoptotic routes [50]. The intrinsic pathway is mitochondria-mediated and mainly regulated by the BCL2 family [51, 52], whereas the extrinsic pathway is triggered through the Fas death receptor, a member of the tumour necrosis factor receptor superfamily [53]. Both pathways converge to a final common path involving the activation of a cascade of proteases called caspases that cleave regulatory and structural molecules, culminating in the death of the cell [54, 55]. To illustrate the mechanisms underlying the suppression effects of AOS on weaning-induced intestinal epithelial cell apoptosis in piglets, the apoptosis-related gene transcriptional levels, including *BAX, BCL2*, *FAS*, *caspase*-*3*, *caspase*-*8* and *caspase*-*9*, were determined. The present study evidenced that AOS ingestion decreased the pro-apoptotic factor *BAX, caspase*-*3* and *caspase*-9 mRNA abundances and increased the anti-apoptotic factor *BCL2* mRNA abundance in the jejunum. Thus, AOS inhibition of intestinal epithelial cell death in weaned pigs might be inclined to decrease mitochondria-dependent apoptosis. Our findings explained the positive role of AOS in rendering the intestinal epithelial cells resistant to weaning-induced apoptosis in piglets.

## Conclusions

To summarise, we indicated that supplementing the diet with 100 mg/kg AOS improved both the intestinal morphology and barrier function and inhibited the enterocyte death by reducing mitochondria-dependent apoptosis in weaned pigs. Furthermore, these changes were accompanied by an enhanced growth performance in weaned pigs. Our observations provide a strong scientific basis for AOS as an alternative to the use of antibiotic growth promoters in swine production and also imply AOS has potential application in clinical nutrition to prevent intestinal disruptions.

## Abbreviations

7-AAD: 7-aminoactinomycin D
ADFI: Average daily feed intake
ADG: Average daily body weight gain
AOS: Alginate oligosaccharide (the basal diet supplemented with 100 mg/kg alginate oligosaccharide)
BAX: B-cell lymphoma-2-associated X protein
BCL2: B-cell lymphoma-2
BW: Body weight
CON: Control (a corn-soybean basal diet)
caspase-3: Cysteinyl aspartate-specific proteinase-3
caspase-8: Cysteinyl aspartate-specific proteinase-8
caspase-9: Cysteinyl aspartate-specific proteinase-9
DAB: 3,3′-diaminobenzidine
G: α-L-guluronic acid
GAPDH: glyceraldehyde-3-phosphate dehydrogenase
G:F: The gain-to-feed ratio
M: β-D-mannuronic acid
MUC1: Mucin 1
MUC2: Mucin 2
MUC4: Mucin 4
PBS: phosphate buffered saline
qPCR: quantitative real-time polymerase chain reaction
sIgA: Secretory immunoglobulin A
